# Prisoners should not be left behind in HCV research and policies

**DOI:** 10.1186/s12954-020-00379-y

**Published:** 2020-05-24

**Authors:** Babak Moazen, Heino Stöver, Kate Dolan, Albrecht Jahn, Florian Neuhann

**Affiliations:** 1 Heidelberg Institute of Global Health, Heidelberg University, Bergheimer Str. 20, Zimmer 317, 69115 Heidelberg, Germany; 2 Department of Health and Social Work, Institute of Addiction Research (ISFF), Frankfurt University of Applied Sciences, Frankfurt/Main, Germany; 3 Program of International Research and Training, National Drug and Alcohol Research Centre, University of New South Wales, Sydney, Australia

## Abstract

With a worldwide prevalence of 15.4%, hepatitis C virus (HCV) has been estimated to be the most prevalent major infectious disease in prisons. The exceptionally high prevalence of HCV in prisons is attributable to common risk behaviors including sharing contaminated tattooing equipment and drug paraphernalia, as well as lack of HCV control interventions including needle and syringe programs. Despite the importance of attention to prisoners as a highly at-risk population to acquire and transmit HCV, the number of HCV research and policy documents ignoring prisoners is increasing. Highlighting this issue, the present manuscript discusses how excluding prisoners from HCV-related research and policies will jeopardize the global HCV elimination goals set forth by the global community.

## Background

At any given time, globally, over 11 million people are held behind bars, while the annual turnover is well over 30 million prisoners [1]. According to a recent estimate, 15.4% of prisoners worldwide are living with HCV [2]. Prevalence of drug injection with shared syringes and drug paraphernalia—the most important risk factor for HCV transmission—in prisons varies between 0.5 and 20.2% in different regions [3]. The highest prevalence rate of unsafe tattooing, another major risk factor for HCV transmission in prison, has been estimated at 45.4% in the Latin American region [3]. In addition to the high-risk behaviors, a lack of HCV control interventions such as needle and syringe programs, as well as diagnosis, treatment, care, and support of HCV, explains the exceptionally high prevalence of HCV in prisons [4].

## Main text

Although prisoners are one of the most vulnerable populations to acquire and disseminate HCV, they have been overlooked in numerous global and regional HCV-related research and policies. One example is a recent global modeling study evaluating the effectiveness of HCV control strategies and the possibility of meeting global HCV elimination targets [5]. The authors focus on very important components of HCV prevention such as implementation of blood safety and infection control, harm-reduction services for injecting drug users, and expansion of screening and treatment, but ignoring prisoners. Another example is a modeling study on the potential prevention benefits of a treat-all hepatitis C treatment strategy at global, regional, and country levels [6], leaving prisoners behind. The third example is a technical report published by the WHO entitled: “Monitoring and evaluation for viral hepatitis B and C: recommended indicators and framework,” again with lack of attention to inmates as a high-risk group [7]. The literature contains numerous other HCV research and policy documents ignoring prison population.

## Conclusion

With over 90% of prisoners eventually being released back to the community, they may act as a vehicle in spreading HCV to the general population. To meet the goals set forth by the global community such as the WHO’s HCV elimination targets and to effectively decrease the global burden of HCV, prisoners need to be included as a key population. HCV prevention is incomplete in the absence of prevention strategies, active case finding, and treatment of positive cases and patients in correctional institutions. The benefits of such programs will go beyond the walls of prisons and affect the general population as well. We are afraid that excluding prisoners from HCV-related research and policies will delay the achievement of the international goals of HCV elimination.

## Data Availability

Not applicable

## Abbreviations

HCV: Hepatitis C virus
WHO: World Health Organization

## References

[1] Moazen B, Assari S, Neuhann F, Stöver H (2019). The guidelines on infection control in prisons need revising. Lancet..

[2] Dolan K, Wirtz AL, Moazen B, Ndeffo-mbah M, Galvani A, Kinner SA (2016). Global burden of HIV, viral hepatitis, and tuberculosis in prisoners and detainees. Lancet..

[3] Moazen B, Saeedi Moghaddam S, Silbernagl MA, Lotfizadeh M, Bosworth RJ, Alammehrjerdi Z (2018). Prevalence of drug injection, sexual activity, tattooing, and piercing among prison inmates. Epidemiol Rev.

[4] Kamarulzaman A, Reid SE, Schwitters A, Wiessing L, El-Bassel N, Dolan K (2016). Prevention of transmission of HIV, hepatitis B &amp; C and tuberculosis in prisoners. Lancet..

[5] Heffernan A, Cooke GS, Nayagam S, Thursz M, Hallett TB (2019). Scaling up prevention and treatment towards the elimination of hepatitis C: a global mathematical model. Lancet..

[6] Trickey A, Fraser H, Lim AG, Walker JG, Peacock A, Colledge S (2019). Modelling the potential prevention benefits of a treat-all hepatitis C treatment strategy at global, regional and country levels: a modelling study. J Viral Hepat.

[7] World Health Organization (WHO). Monitoring and evaluation for viral hepatitis B and C: recommended indicators and framework. Geneva: World Health Organization (Global Hepatitis Program); 2016. Available from: https://apps.who.int/iris/bitstream/handle/10665/204790/9789241510288_eng.pdf?sequence=1 [Access date: 23 April 2020].

